# Prevalence, distribution and risk factors for brucellosis infection in goat farms in Ningxiang, China

**DOI:** 10.1186/s12917-021-02743-x

**Published:** 2021-01-19

**Authors:** Yin Li, Dan Tan, Shuang Xue, Chaojian Shen, Huajie Ning, Chang Cai, Zengzai Liu

**Affiliations:** 1 Changsha Animal Disease Control Center, No.12 Xianjiahu Road, Changsha, 410007 Hunan P.R. China; 2 Murdoch University, Murdoch, 6155 WA Australia; 3 Commonwealth Scientific and Industrial Research Organisation, St Lucia, 4067 QLD Australia; 4 Animal Health and Epidemiology Center, No. 369 Nanjing Road, Qingdao, 266032 Shandong P.R. China; 5 Zhejiang A&F University, Hangzhou, 311300 China

**Keywords:** Brucellosis, Risk factors, Goat farming, Ruminants, China

## Abstract

**Background:**

In south China, goats are the major source of Brucellosis for human infection. However, there are few studies on the prevalence of and risk factors for goat brucellosis in south China. In this study, we conducted a cross-sectional study to investigate the herd prevalence, spatial distribution and relevant risk factors for goat brucellosis in Ningxiang county, south China. Commercial goat farms (*n* = 457) were randomly selected, and their disease status was ascertained by testing serum samples of chosen individuals using the Rose Bengal Test (screening test) and the Serum Agglutination Test (confirmatory test) in series. A farm with at least two positive individuals was defined as a case farm. Standardized questionnaires were used to collect information on management and hygiene practices in farms. A logistic model with a binomial outcome was built to identify risk factors for being seropositive.

**Results:**

The true herd prevalence in commercial goat farms was 4.5% (95%CI: 0.2%-12.2%) and the townships in the centre of the county had higher herd prevalence. The risk factors associated with seropositive on local goat farms include “Introduction in the past 12 months” (OR= 61, 95%CI: 16-333), “Improperly disposal of the sick or dead goats” (OR= 33, 95%CI: 5-341) and “Poor hygiene in lambing pen” (OR= 25, 95%CI: 5-192).

**Conclusions:**

These findings will aid in the development of control strategies of Brucellosis in south China and risk factors identified in this study should be taken into consideration when designing a control strategy.

## Background

Brucellosis is a zoonotic disease caused by the genus *Brucella spp* [1]. It can cause large economic losses from abortion in livestock and serious syndromes in humans [2–4]. In human patients, the typical clinical symptoms include fever, fatigue, hyperhidrosis, and joint pain [5]. Brucellosis is an occupational disease and the most relevant occupations are livestock farmers, veterinarians, and workers in butcher shops, the milking and dairy processing industries [6]. People are infected following contact with infected animals and the consumption of contaminated animal products, including unpasteurized milk and raw meat [7].

Brucellosis is endemic in many countries in the Mediterranean Basin, the Middle East and South America [8]. There were 37,947 incidences of human Brucellosis diagnosed in China in 2018 [9]. Several studies had reported that *B. melitensis* was the dominant species isolated from patients in China, although *B. abortus* and *B. suis* also prevail in certain provinces (Sichuan, Guangxi and Guangdong, respectively, [10–12]).

Brucellosis was well-controlled in China before 1980, via vaccination and other measures, and human cases were mainly seen in northern provinces, namely, Inner Mongolia, Xinjiang, Tibet, Qinghai, and Ningxia [10, 13]. However, Brucellosis has remerged since the 1990s, and the affected area expanded from northern pastureland provinces to the adjacent grassland and agricultural areas, then to southern coastal and southwestern areas [13]. Since 2010, significant increases in human cases have been detected in south China [11, 13, 14]. For example, among the newly infected counties in 2016, 90% of them were in south China [15].

In south China, goats are more commonly raised than sheep [16], thus are the potential major source of Brucellosis for human infection in this area [17]. However, there are few studies on the prevalence and risk factors of Brucellosis in goat farms in south China. In this study, we investigated the herd prevalence, spatial distribution and relevant risk factors for goat brucellosis in Ningxiang county, south China. These findings will aid in designing control strategies of Brucellosis in south China.

## Results

### Herd‐level prevalence of infection

In Ningxiang county, the apparent and true herd prevalence in commercial goat farms were 4.8% (95%CI: 3.0%-7.2%) and 4.5% (95%CI: 0.2%-12.2%). In the infected commercial farms, the crude individual prevalence was 43.7% (40.0–47.0%) in general.

The detailed test results for each town shows that the apparent herd prevalence varies among different towns. See Table 1.

**Table 1:** Herd prevalence of Brucellosis in townships in Ningxiang county

Township	Commercial farms			
	Total farms	Sampled farms	Positive farms	Prevalence
Dachengqiao	13	11	1	9% (2-16%)
Hengshi	25	10	7	70% (35-93%)
Huaminglou	22	13	1	7% (0-17%)
Huilongpu	16	13	1	7% (1-14%)
Shuangfupu	27	27	5	19%
Huangcai	61	61	1	1.6%
Laoliangcang	25	25	1	4%
Yujiaao	48	48	5	13%
Zifu	19	19	1	5%
Weishan	15	15	0	0
Datunying	18	11	0	0
Donghutang	14	9	0	0
Fengmuqiao	8	4	0	0
Huitang	8	8	0	0
Jinzhou	6	6	0	0
Qinghuapu	7	7	0	0
Meitanba	14	14	0	0
Nantianping	5	5	0	0
Qingshanqiao	26	26	0	0
Shantian	15	8	0	0
Shuangjiangkou	5	5	0	0
Xiaduopu	5	5	0	0
Zhuliangqiao	2	2	0	0
Longtian	6	6	0	0
Batang	14	14	0	0
Lijingpu	11	11	0	0
Xieleqiao	19	19	0	0
Xiangzikou	31	31	0	0
Chengjiao	6	6	0	0
Daolin	18	18	0	0

### Spatial distribution of goat brucellosis

**Fig. 1:**
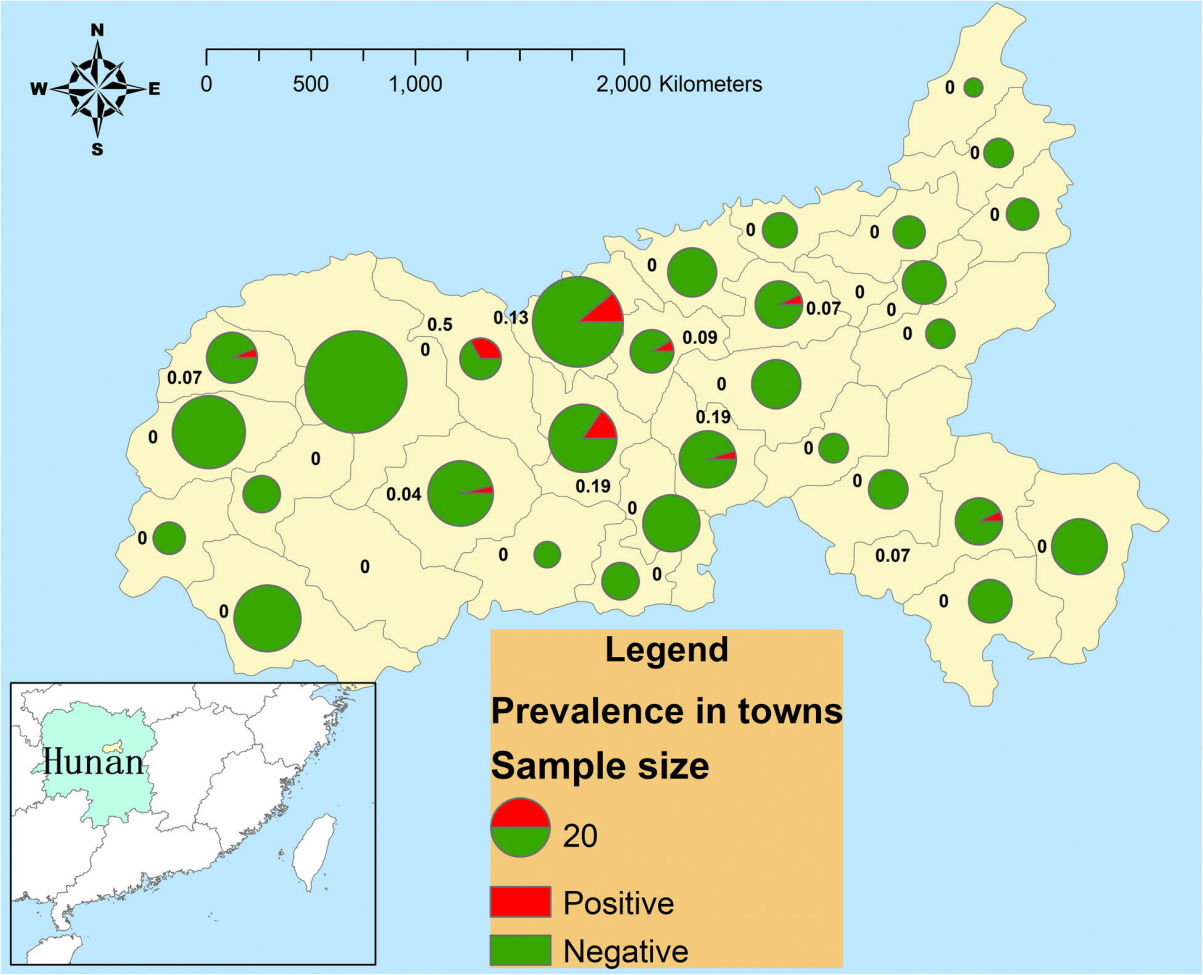
Herd prevalence in towns in Ningxiang County. The image depicted in this figure was created by the authors

Nine out of 30 townships had goats that were positive for Brucellosis. (See Table 1). The commercial farms in the townships in the centre of Ningxiang County had higher herd prevalence. See Fig. 1.

### Univariate logistic regression analysis

Nine potential risk factors were identified by univariate logistic regression analysis (*p* < 0.2). Among these factors, introduction, self-breeding, and safe disposal of sick or dead animals were the factors with the strongest association with disease present. See Table 2.

**Table 2:** Results of the univariable logistic regression for risk factors for Brucellosis in goat farms in Ningxiang county

Factor	Categories	No. +ve/total (%)	OR	95% CI		***P***-value
				Lower	Upper	
Introduced in past 12 months	Yes	20/46 (43)	42.05	13.29	187.67	<0.01
	No	3/167 (2)				
Whether clean and disinfect the lambing pen or field	Yes	9/125 (7)	0.41	0.16	0.98	0.05
	No	14/88 (16)				
Whether have other species on farm	Yes	1/29 (3)	0.26	0.01	1.33	0.20
	No	22/184 (12)				
Whether have quarantine field	Yes	1/34 (3)	0.22	0.01	1.09	0.14
	No	22/179 (12)				
Whether have separated lambing pen or field	Yes	1/81 (1)	0.06	0.01	0.31	<0.01
	No	22/132 (17)				
Whether conduct disinfection on visitors	Yes	16/201 (8)	0.14	0.01	0.70	0.06
	No	7/12 (58)				
At least disinfect the field once every week	Yes	2/96 (2)	0.10	0.02	0.34	<0.01
	No	21/117 (18)				
Self-breed	Yes	15/201 (7)	0.04	0.01	0.14	<0.01
	No	8/12 (67)				
Whether bury the sick or dead goat	Yes	16/201 (8)	0.06	0.02	0.21	<0.01
	No	7/12 (58)				

### Multivariate logistic regression analyses

There is no significant clustering of farm-level data at these levels. Interactions between factors in the final model were checked, and none of them improves the model. A multivariate logistic regression model was built with three risk factors included (Table 3). Hosmer-Lemeshow test indicates a good fit of the model to the data (*P* = 0.88) and the area under the ROC curve (AUC) is 0.92 (95%CI:0.87–0.97).

**Table 3:** Results of the multivariable logistic regression for risk factors for Brucellosis in goat farms in Ningxiang county

	β	SE	OR	95% CI for OR	
				Lower	Upper
Introduced in the past 12 months	4.12	0.76	61.4	15.8	332.7
Bury the sick or dead goats	3.49	1.05	32.9	5.0	340.7
Not clean and disinfect the lambing pens or field	3.21	0.89	24.7	5.2	192.0
Constant	0.53				

## Discussion

In recent years, the incidence of Brucellosis has been increasing in Southern China, especially in Hunan, Zhejiang, Guangdong, Yunnan, Jiangsu provinces [7]. Some studies concluded that control brucellosis in livestock is the key to mitigate the risk of human infection [18, 19]. Thus, understanding the epidemiology of Brucellosis in goat farms in south China is highly demanded. To our knowledge, this is the first study to investigate the herd-level prevalence of Brucellosis and associated risk factors in goat farms in south China.

The infection of Brucellosis in goat farms is a significant threat to public health and livestock industry in south China. In this study, it was evaluated that 4.5% of goat farms in Ningxiang had brucellosis infection in 2015. These infected farms may have led to an increasing number of local human cases. Up to 2016, more than 60 human cases were diagnosed in this county since 2010, when the first case was reported. Most of them were goat farmers and slaughter workers (personal communication with officers from Ningxiang CDC). Nevertheless, the disease in goat farms can cause a significant economic loss to local farmers. The consequences from brucellosis infection include abortion, stillbirths and increased mortality in goats [20]. A study in India reported a loss of 0.5 USD per goat due to brucellosis infection [21]. In addition, surveillance on Brucellosis is resource-consuming [22]. In China, the control of Brucellosis in livestock requires intensive inputs on activities like collecting samples in the field, testing, culling, disposal and compensation to farmers [23].

Several risk factors for having seropositive goats in local goat farms were identified in this study. A goat farm having an introduction in the preceding year would have a dramatically increased risk of infection (OR = 61) than a farm without introduction in the preceding year. This finding indicates that there might be risky trade practices adopted by local farms. According to the local official veterinaries, local goat farmers tend to purchase goats from local livestock markets or even from northern provinces, because the price of these goats is often lower than the price of local goats. Few farmers would have a concern about Brucellosis when they purchase goats from other provinces. Besides, improperly dispose of the sick or dead goats is another risky practice adopted by local farmers (OR = 33). The most practical way of dealing with the dead goats would be burying the carcasses. However, local farmers often feed the contaminated carcasses to dogs or abandon them carelessly. The bacteria could survive for months in filth [24]. Thus, the indirect transmit may occur through the contacts between dogs and contaminated soil and water or vectors [24, 25]. The other risk factor identified in this study is the poor hygiene in lambing pen (OR = 25), where has a higher chance to be contaminated by abortions. Our findings agree with studies in other countries [26–28]. These findings may explain why there was a big increase in the human case in south China since 2010 [7, 13]. We suggest that the animal health authorities in south China should enhance quarantine on the imported ruminants from northern provinces. Health certification should be required for the imported goats. Subside is offered to farmers for taking safe disposal on carcasses in several provinces in China [29]. However, Authorities should also establish sufficient disposal facilities to ensure the contaminated fetus could be processed properly. Besides, sufficient education to farmers is needed in south China. Especially, local farmers should be taught on how to improve hygiene in lambing pens.

Brucellosis in sheep and goats is contributing to local human infection. A study revealed that the distribution of human cases was significantly more spatially correlated with the number of sheep and goats than with swine and cattle [30]. Control of Brucellosis in human requires a good control of Brucellosis in livestock. Unfortunately, there has been no reliable strategy of brucellosis control in many developing countries [2]. Understanding of the epidemiology of goat brucellosis in south China is the key to the development of efficient control strategy for brucellosis control in China, and risk factors identified in this study should be taken into consideration when designing a control strategy. For example, the introduction was found to be the most relevant risk factor for infection in local goat farms. Thus future control strategy should emphasize on quarantine during the introduction. Besides, the local veterinary and public health authorities should promote education to local goat farmers to improve their awareness of testing brucellosis in goats before introduction. To eliminate Brucellosis in local farms, “screen and cull” strategy is implemented in many places in China [19, 31]. The model established in this study shows good fitness in predicting cases and could be used to guide a risk-based sampling in the field.

The spatial distribution was heterogeneous among townships in Ningxiang County. Nearly 30% of townships in this county had cases. This indicates that Brucellosis can spread widely in a county. The townships in the centre of the county had a higher herd prevalence than the other townships. The reasons might be that these townships had larger goat populations and higher goat density (personal communication), and they are closer to the downtown area of the county than the other townships. Goat farmers in these towns may have greater access to the livestock markets because the livestock markets are often located in suburban areas (personal communication with experts from local veterinary officers). The goat farms near the suburban areas may have more frequent connections with markets; thus they have a higher risk of introducing Brucellosis from markets. It is worth noting that almost all the human cases were found in these towns in recent years (personal communication with experts from local veterinary officers). We suggest that animal health authorities in south China should be aware that Brucellosis can spread widely in an area. More studies on the role of livestock markets in the spread of Brucellosis in south China are needed.

High between-herd prevalence of Brucellosis has been reported in other Asia countries. A cross-sectional survey in Jordan reported 45.4% (95% CI: 30.3–61.6) in goat herds and 70.4% (95% CI: 55.5–84.9) in mixed sheep-goat flocks [28]. Herd prevalence of Brucellosis in goat farms in China had been reported with lower values than that. A cross-sectional study in Dalian city, Liaoning province, northeast China, reported 8.4% (95% CI: 4.4–12.5) herd prevalence in local goat farms. Moreover, they estimated herd prevalence in commercial goat/sheep farms was 13.3% (95% CI: 5.6–21.0), while herd prevalence in backyard farms was 8.2% (95% CI: 5.2–11.2). Our study reported a lower herd prevalence (4.5%) in commercial goat farms in Ningxiang County in 2015. However, the prevalence may have been increasing since then, as Brucellosis is expanding to south China [7].

There were limitations in this study. One thing worth pointing out is that the crude individual prevalence in infected farms was likely overestimated in this study, due to the risk-based sampling strategy for onsite individual selection. The Rose Bengal Plate test (RBPT) and the Serum Agglutination Test (SAT) were used in serial as the diagnostic tests in the field, as they were recommended diagnosis tests in *the national surveillance plan for major animal diseases (2015)* [32]. However, the low specificity of the SAT would lead to false-positive results. We defined a farm with at least two positives as a case farm to address this problem. Other tests with better specificity, such as the complement fixation test, the fluorescence polarization assay and enzyme-linked immunosorbent assays should be used as the confirmation tests in the surveillance programs on Brucellosis in small ruminants in China [33]. The response rate to the questionnaire was 46.6% in this study, due to the fact that it was voluntary for the farmers to participate in the interview or not. This may have introduced bias in this study. Incentive, such as gifts or subsides, should be offered in future studies to encourage farmers to participate.

## Conclusions

The herd prevalence of Brucellosis in goat farms in Ningxiang county was evaluated and the spatial distribution of brucellosis infection in townships was described in this study. Introduction and poor hygiene in local goat farms were key risk factors for local farms having goats that were seropositive to Brucellosis. A polished control strategy should further target to promote quarantine during the introduction, safe disposal and good hygiene in lambing pens on the farm.

## Methods

### Study design and study population

A cross-sectional survey was conducted in all the 30 townships in Ningxiang County between June and October 2015. The study unit was a goat farm, and the study population were all the commercial (greater than 30 goats) goat farms in Ningxiang County.

### Sampling strategy

Commercial herds were selected for inclusion in the study by stratified random sampling. Before this survey, a survey on the total number of commercial goat farms was conducted by local Centre for Animal Diseases Control (CADC) in April 2015 in order to record all the commercial goat farms to create a sampling frame. The study population was stratified by the township. In 22 out of the 30 townships, all the commercial goat farms were sampled, while in the other 8 townships, the farms with more than 30 goats were randomly selected within the listed farms in each township. A random number generator was used to select farms. A census on all the commercial farms in the 8 townships was not possible due to financial resource limitation in these townships. The proportion of the sampled farms over the total farms were range from 50–85% in these townships. During the study period, 457 farms were sampled in total.

### Sample size

Inside a farm, individuals were risk-based selected. Only the goats older than 6 months were involved because they have a higher susceptibility to Brucellosis [27]. All the female goats with known abortion history were chosen when sampling on a farm. For selecting the other goats without known abortion history, systematic random sampling was used. Goats were forced to walk through a narrow gate one by one to get their orders, and the ones were picked with a fixed interval. For example, if 5 goats were sampled from 100 goats, we picked a random number (10 for instance) from 1 to 20, then we sampled the 10th, 30th, 50th, 70th, and 90th of the goats that went through the gate.

A serial testing strategy was used to ascertain farm infection status, combining RBPT and the SAT. Based on estimates in published literature and combining with expert opinion (personal communication with experts in the laboratory of China Animal Health and Epidemiology Center), sensitivity (Se) and specificity (Sp) values for the RBPT are: 0.9 ≤ Se ≤ 1; 0.85 ≤ Sp ≤ 0.95 and for SAT: 0.8 ≤ Se ≤ 0.95; 0.95 ≤ Sp ≤ 1 [28, 34–39]. The minimal sample size required to reach certain confidence of detecting at least one positive animal was calculated with software ProMESA version 2.3.0.2( INTA&Massey University, Castelar, Argentina) with a scenario: The fixed value of Se and Sp values were used, with RBPT are: Se = 0.95; Sp = 0.9 and for SAT: Se = 0.9; Sp = 0.97; At least 40% seroprevalence of Brucellosis within an infected goat farm; The probability of detecting at least one animal if the herd is infected was set as 90%; Herd sizes of goat farms was set as 60 because the averaged size of local commercial goat farms was 60 animals. The formula used for sample size calculation is as follows:

$$ \mathrm{n}=\left[1-{\left(1-\mathrm{CL}\right)}^{1/e}\right]\times \left(\mathrm{N}-\frac{e-1}{2}\right) $$

$$ \mathrm{e}=\mathrm{N}\times \mathrm{p}\times \mathrm{Cse} $$

where n is the sample size required in a farm; CL is the level of confidence; e is the number of detectable cases in an infected farm; p is the designed individual prevalence in an infected farm; N is the average farm size; Cse is the combined sensitivity of the two tests [40]. RBPT and SAT were used in series in this study; thus, the combined sensitivity (Cse) and specificity (Csp) were: Cse = 0.86 and Csp = 0.99. The calculation formula for the combined sensitivity and specificity of two tests can be found on the website of Ausvet [41]. The result from these calculations suggested that at least 5 goats would be sufficient to reach the desired probability of detection under the assumptions above. In the field, 8 goats were sampled on a farm on average.

### Data collection

Five ml blood was collected from the jugular vein of each goat into plain vacutainer tubes and kept cold during transport to the laboratory. The serum was removed after centrifugation and stored at 4 °C until testing.

Serum samples were initially screened with RBPT using commercial antigens (YEBIO, China). Samples positive to RBPT were confirmed with the SAT (YEBIO, China). All serological tests were performed, and results were interpreted according to the national standard procedures. A standard questionnaire was used to collect information from selected goat farms. The major questions included in the questionnaire were listed in Table 4.

**Table 4:** The main contents of the questionnaire

Categories	Questions
Basic producer information	How many goats on the farm?
	Are the goats ranged all the time?
	Any other species on the farm?
	Self-breeding or not?
Introduction	Did you introduce in the past 12 months?
	Did you sell any goats in the past 12 months?
	Whether have quarantine field on the farm?
Biosecurity practices	Whether clean and disinfect the lambing pen or field?
	Whether have separated lambing pen or field on the farm?
	Do you ask visitors to clean and disinfect their clothing/boots before they enter the farm?
	How often do you disinfect the pens?
	What do you do with the sick or dead goats?

All the owners of the sampled farms were invited to accept an interview with the questionnaire, although the response to the questionnaire was voluntary. A total of 213 farms completed the questionnaire. The survey was part of *the national surveillance plan for major animal diseases, 2015*, and all the activities follow the ethics requirement of the plan.

### Data analysis

A goat with positive results in both RBPT and SAT tests was considered as infected, and a farm with at least two infected goats was considered as an infected farm.

The apparent herd prevalence for commercial farms was calculated considering sample weight in each stratum (township) in Microsoft Excel (Redmond, WA, USA) using the method of Dohoo, Martin et al. (page 35–37, [42]). Herd sensitivity (HSe) and specificity (HSp) were calculated as described by another study [28]. The formulae were as following:

$$ \mathrm{HSe}=1-{\left(1-{\mathrm{AP}}_{\mathrm{POS}}\right)}^{\mathrm{n}} $$

$$ {\mathrm{AP}}_{\mathrm{POS}}=\mathrm{P}\times \mathrm{CSe}+\left(1-\mathrm{P}\right)\ \left(1-\mathrm{CSp}\right) $$

$$ \mathrm{HSp}={\mathrm{CSp}}^{\mathrm{n}} $$

where P is the individual prevalence in positive goat farms, CSe is the combined sensitivity of the two tests used, CSp is combined specificity of the two tests and n is the number of tested animals in one goat farm. The true seroprevalence at farm level was calculated after adjusting for HSe and HSp as TPH= (APH+ HSp − 1)/(HSe + HSp − 1). To address the variability and uncertainty in the performance of the diagnostic tests at the individual animal level and the effect of the within-herd sampling fraction, Monte-Carlo simulation approach used by a study [28] was implemented using basic R (3.0.2). The CSe was set as a range of value between 0.85 and 0.99 that follow a uniform distribution, CSp was set as a range of value between 0.95 and 0.99 that follow a uniform distribution, the P was set as a range of value between 0.35 and 0.45 that follow a uniform distribution, and n was a randomly selected number from the real values of sample sizes in goat farms in each iteration. The 95% confidence interval for the estimated seroprevalence at herd levels was obtained from the output of a simulation of 10,000 iterations.

We also calculated crude individual prevalence in the infected farms, using total positives divided by the total number of tested in positive commercial farms.

A Map was developed with ArcGIS 9.3 (ESRI Inc., Redlands, CA, USA) to show the study area and the values of herd prevalence in townships.

Univariate and multivariate logistic regression analysis was conducted on the data from 213 interviewed farms, using the software SPSS (SPSS version 19, Inc., IBM Corporation, Somers, NY) and R package lme4 [43]. In order to avoid false positives, the definition of a case farm was one with at least two goats positive with both RBPT and SAT tests. For multivariate logistic regression analysis, all the factors with P-values less than 0.2 in the univariate logistic regression analysis were used to build a multivariable model using a stepwise backward method. A variable was retained when the P-value of the likelihood ratio test was less than 0.05. Linear Mixed-Effects Models was built with village and township as the random effect to adjust for clustering of farms within villages and townships. The fitness of the final model was tested by Hosmer-Lemeshow test. The area under the ROC curve was calculated with R package pROC [44] and SPSS.

## Data Availability

The data used in this study is under the regulation of the Key Project of Modern Agriculture Techniques in Changsha. It will be available from the corresponding author.

## Abbreviations

AUC: The area under the receiver operating characteristic curve
CADC: Centre for Animal Diseases Control
Cse: Combined sensitivity
Csp: Combined specificity
HSe: Herd sensitivity
HSp: Herd specificity
RBPT: Rose Bengal Plate Test
SAT: Serum Agglutination Test
Se: Sensitivity
Sp: Specificity
